# A stress test to evaluate the usefulness of Akaike information criterion in short-term earthquake prediction

**DOI:** 10.1038/s41598-020-77834-0

**Published:** 2020-12-03

**Authors:** Roberta Tozzi, Fabrizio Masci, Michael Pezzopane

**Affiliations:** 1 Istituto Nazionale di Geofisica e Vulcanologia, Rome, 00143 Italy; 2 Istituto Nazionale di Geofisica e Vulcanologia, L’Aquila, 67100 Italy

**Keywords:** Natural hazards, Space physics, Seismology

## Abstract

Akaike information criterion (AIC) has been recently adopted to identify possible earthquake precursors in ionospheric total electron content (TEC). According to the authors of this methodology, their technique allows finding abrupt increases (positive breaks) in vertical TEC rate of change 25–80 min before the occurrence of large earthquakes, highlighting a promising implication of AIC method in Mw > 8 earthquakes alert strategies. Due to the relevance of this matter, a lively scientific debate ensued from these results. In this study, we carefully evaluate AIC method potentiality in searching earthquake TEC precursory signatures. We first investigate the dependence of the detected breaks number on the adjustable AIC method parameters. Then, we show that breaks occurrence clusters around specific local times and around moderate and high solar and geomagnetic activity. The outcome of this study is that AIC method is not concretely usable for issuing large earthquakes alerts.

## Introduction

The search for precursory signals of earthquakes is aimed toward the monitoring that could allow predicting the occurrence of the earthquake, someday. Despite the great economic and scientific effort, and the publication of numerous papers reporting the observation of pre-earthquake anomalies in ionospheric data^1–6^, to date we are not yet able to predict location, time and magnitude of an incoming earthquake. An effective method for earthquake prediction requires the identification of reliable and reproducible precursory signals and low false alarm rates. Any method candidate for identifying precursors should be supported by concrete evidence of its reliability^7–15^.

Many papers report of earthquake-induced ionospheric effects in total electron content (TEC) observed shortly after the shock. These coseismic ionospheric disturbances generated by the earthquake are well understood and described in scientific literature^16–19^. Differently, a direct correlation between the observed TEC changes and an incoming earthquake has still to be validated by the scientific community. Recent studies^10,12,20–24^ have rather shown that many observed pre-earthquake TEC changes were not associated with the incoming earthquake but were actually driven by either changes in solar and geomagnetic activities, or artifacts related to the adopted procedure of analysis.

Among the authors reporting pre-earthquake changes in ionospheric data, Heki^3^ and Heki and Enomoto^4^ analyzed, respectively, slant TEC (sTEC) and equivalent vertical TEC (vTEC) recorded around Mw > 8 earthquakes. These studies showed the occurrence of an anomalous TEC increase that systematically starts $\sim$ 40 min prior the shock, suggesting a relation with the earthquake, and then a possible implication in short-term prediction. What reported by Heki^3^ and Heki and Enomoto^4^ has been seriously questioned by Masci et al.^23^, Kamogawa and Kakinami^25^ and Utada and Shimizu^26^. According to Masci et al.^23^, the pre-earthquake TEC increase identified by Heki^3^ and Heki and Enomoto^4^ should be regarded as an artifact induced by their analysis rather than a precursor of the incoming earthquake (see Supplementary Discussion S1 for details).

Following the above-mentioned criticalities, Heki and Enomoto^27^ proposed a new numerical approach based on Akaike information criterion^28^(AIC) to detect abrupt increases (positive breaks in their terminology) in the vTEC rate of change shortly before earthquakes, associating them to the onset of pre-earthquake anomalies. Considering the extreme relevance and complexity of this matter, and the potential societal relevance of the results found by Heki and Enomoto^27^, i.e. the possibility to be able to detect signs of an incoming strong earthquake in real-time monitored vTEC data, is of uttermost importance to rigorously check the reliability of their results.

Our idea is to reproduce their work and undergo AIC method a stress test to assess its potentiality for short-term earthquake prediction or early warning. We consider four Mw > 8.0 earthquakes addressed by Heki and Enomoto^27^, but differently from them we use a much wider vTEC database to build a more reliable and consistent statistics: one solar year (the year of the specific earthquake) of vTEC values also far from the earthquake instead of a few months around it. The four earthquakes are: 2011 Tohoku-Oki (Mw 9.1), 2012 North Sumatra mainshock (Mw 8.6), 2012 North Sumatra largest aftershock (Mw 8.2) and 2014 Iquique (Mw 8.2) (see Table 1 for the main characteristics of each earthquake).

**Table 1:** Main parameters of the four $\hbox {Mw}$ > 8.0 examined earthquakes.

Earthquake	Country	Coordinates	Mw	Date origin time (UT)	**Hypocentral depth (km)**
Tohoku-Oki	Japan	37.52$^\circ$ N 143.05$^\circ$ E	9.1	2011-03-11 5:46:23	14.5
North Sumatra mainshock	Indonesia	2.327$^\circ$ N 93.063$^\circ$ E	8.6	2012-04-11 8:38:37	14.5
North Sumatra largest aftershock	Indonesia	0.802$^\circ$ N 92.463$^\circ$ E	8.2	2012-04-11 10:43:09	25.1
Iquique	Chile	19.642$^\circ$S 70.871$^\circ$W	8.2	2014-04-01 23:46:50	20.1

Since a first step of our work is to reproduce the results found by Heki and Enomoto^27^, it is fundamental to work with data at best matching theirs. So, for each earthquake, we select GPS measurements, with a time sampling of 30 seconds, from the same pairs receiver-satellite. vTEC has been derived using the method by Ciraolo et al.^29^, details are reported in the Supplementary Discussion S2. Table 2 lists the GPS receiver-satellite pairs we consider.

**Table 2:** GPS satellite-receiver pairs used to estimate vTEC.

Earthquake	Receiver	Satellite
Tohoku-Oki	GEONET 3009	15
	GEONET 0221	26
North Sumatra mainshock	SuGAr LHW2	32
	SuGAr BNON	32
North Sumatra largest aftershock	SuGAr LEWK	20
	SuGAr UMLH	32
Iquique	IGS AREQ	20
	IGS IQQE	23

Differently from Heki and Enomoto^27^ we let the adjustable parameters of AIC method vary, instead of *a priori* setting them differently for each case study. We also investigate the possibility that seemingly anomalous ionospheric perturbations might actually be normal variations driven by the Sun-Earth interaction and study a possible dependence on local time and solar and geomagnetic activity.

We will start describing the AIC method and illustrating results from the validation of our own algorithm. Then outcomes from the application of AIC method to selected data will be illustrated and discussed.

We mention that although Heki and Enomoto^27^ is presented by the authors themselves as a paper to demonstrate the baselessness of the criticisms made by Masci et al.^23^, its core is instead the application of AIC to identify possible precursory signals of the earthquake in TEC data. Supplementary Discussion S1 also reports some comments on the Heki and Enomoto’s^27^ attempt to reply to the issues raised by Masci et al.^23^.

## AIC method: description and algorithm validation

AIC is a concept taken from information theory to provide an estimate of the information that would be lost if a particular model were used to describe the process that produced the analyzed data. So, through AIC, it is possible to compare the quality of a set of statistical models to each other and rank them from the best to the worst, depending on the amount of the information lost on the process under investigation. It is important to underline that AIC can be used only in a comparative way, not to establish the absolute goodness of a model. In formula AIC is written as follows:

1 $$\begin{aligned} {\mathrm {AIC}} = {n}\cdot {\mathrm {ln}}(\sigma ^2)+2{k} \end{aligned},$$

*n* is the number of data, $\sigma ^2$ the sum of squared residuals for the model under evaluation, and *k* the number of its free parameters. From Eq. (1) it follows that the best model is the one characterized by the lowest AIC since it is the one with the lowest value of $\sigma ^2$, and hence the one that best represents the process under investigation.

**Figure 1:**
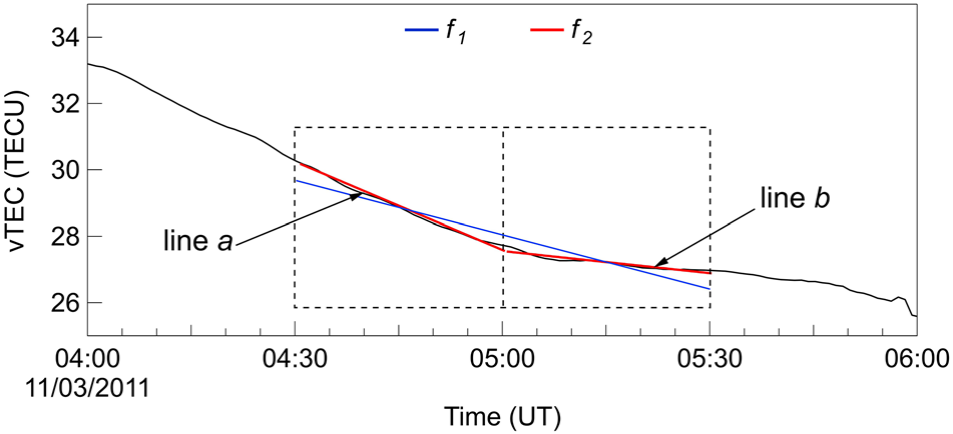
Example of AIC method functioning. vTEC (black curve), linear fit $f_1$ (blue line), piecewise linear fit $f_2$ (red line). Dashed black rectangle indicates the time window over which fitting is made, in this case its width $\Delta t$ is of 60 min.

For instance, given two possible models, the relative test of model quality is made computing the difference between the AIC for each model, i.e. $-\Delta \mathrm {AIC}$ is computed.

This method, however, can be used also in a reverse way, for instance when looking for a particular behavior in a time series. In this case two models must be chosen: one that describes exactly the behavior one is looking for and the other describing a different behavior. In this way, when the analyzed time series will display the desired behavior, its associated AIC will be lower than that associated to the other model.

Heki and Enomoto^27^ used this criterion exactly in this way to find changes in the rate of change of vTEC, specifically V-shaped variations in the slope of vTEC when displayed against time. To achieve this goal, they compared two models: a linear model and a piecewise linear model that reconstructs V-shaped changes (see Fig. 1). In the case of the linear fit ($f_1$), the number of free parameters *k* of Eq. (1) is equal to 2, while in the case of the piecewise fit ($f_2$) *k* is equal to 3 (for two distinct linear fits *k* = 4 but the constraint of continuity of the fitting function removes one degree of freedom).

To better explain the method used by Heki and Enomoto^27^, Fig. 1 displays vTEC data (black curve) together with the two models (red and blue lines) used to represent them. In this example the window over which the fit is performed (black dashed rectangle) has a size $\Delta t$ = 60 min, extending from 30 min before to 30 min after the center of the fitting window. Since vTEC is given with a time resolution of 30 seconds, the number *n* of points falling in this window is equal to 121. Let us suppose to compare the performance of the two models $f_1$ and $f_2$ over the time interval covered by the fitting window. Once estimated $f_1$ and $f_2$, the corresponding values of $\sigma ^2$ can be calculated and hence that of $-\Delta {\text{AIC}}= {\text{AIC}}_{f_2} - {\text{AIC}}_{f_1}$, that will assess which between the two models best represents vTEC data within the fitting window. The better the model the lower the associated $\sigma ^2$ and AIC value so, when the behavior of vTEC displays a V-shape, the piecewise fit ($f_2$) will much better model vTEC data than the linear fit ($f_1$) thus resulting in positive values of $-\Delta \mathrm {AIC}$: what Heki and Enomoto^27^ call positive a break. An amplitude is associated to each positive break, defined as the difference between the angular coefficients of the lines that fit vTEC data in the first half (line a of Fig. 1) and in the second half (line b of Fig. 1) of the fitting window. Moreover, since Heki and Enomoto^27^ are interested only in detecting those vTEC V-shaped behaviors representing an increase in the vTEC rate of change, they assigned a null value whenever either $-\Delta \mathrm {AIC}$ returns negative values (meaning that vTEC has a linear behavior) or the V-shape is indicative of a decrease in the vTEC rate of change.

Moving the fitting window in time of 1 point, it is possible to build a $-\Delta \mathrm {AIC}$ time series by associating to each value the time corresponding to the center of the fitting window. Similarly, a time series of the amplitudes of positive breaks can be reconstructed. There is not a precise rule to choose the size $\Delta t$ of the fitting window. Certainly it must have a length such to guarantee a sufficient statistics for $-\Delta \mathrm {AIC}$ estimation but also a good time resolution of $-\Delta \mathrm {AIC}$ time series. If the AIC method is applied to vTEC time series as simply as explained above, a large number of positive breaks will be found. So, to make a sort of selection and keeping only the most intense breaks Heki and Enomoto^27^ introduced two thresholds for breaks amplitude: the absolute and relative thresholds ($Th_{\mathrm{A}}$ and $Th_{\mathrm{R}}$, respectively). The absolute threshold $Th_{\mathrm{A}}$ corresponds to a minimum value (expressed in TECU/h) that break amplitude must reach to be “significant”. Similarly, the relative threshold $Th_{\mathrm{R}}$ corresponds to a minimum value that the ratio (expressed in %) between the slopes of lines a and b of Fig. 1 must reach to be “significant”. Heki and Enomoto^27^ do not explain the procedure to set these thresholds in real time monitoring but they anyway choose specific values of $\Delta t$ , $Th_{\mathrm{A}}$ and $Th_{\mathrm{R}}$ for the different earthquake case studies.

**Figure 2:**
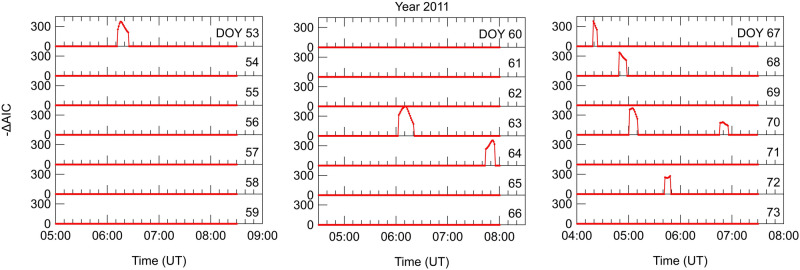
Results from AIC algorithm validation. $-\Delta \mathrm {AIC}$ daily plots from 22 February 2011 (DOY 53) to 14 March 2011 (DOY 73). This figure represents a reproduction of Fig. 6 (panels d, e, and f) by Heki and Enomoto^27^ but obtained with our own AIC algorithm using the same values of $\Delta t$ , $Th_{\mathrm{A}}$ and $Th_{\mathrm{R}}$ ($\Delta t$ = 60 min, $Th_{\mathrm{A}}$ = 3.5 TECU/h, $Th_{\mathrm{R}}$ = 75%). vTEC data used for this figure are derived from measurements made by the GEONET receiver 3009 related to the GPS satellite 15.

Before applying AIC method to the extended datasets, we validate our AIC algorithm on vTEC analyzed in Heki and Enomoto^27^ to investigate the presence of positive breaks preceding the 2011 Tohoku-Oki earthquake. Figure 2 (panels a, b and c) reproduces Heki and Enomoto’s^27^ Fig. 6 (panels d, e and f); it displays $-\Delta \mathrm {AIC}$ from 22 February 2011 to 14 March 2011 calculated with our algorithm applied on vTEC data derived from measurements made by the GEONET receiver 3009 related to the GPS satellite 15. We use the same values of $\Delta t$ , $Th_{\mathrm{A}}$ and $Th_{\mathrm{R}}$ used by Heki and Enomoto^27^ for their Fig. 6: $\Delta t$ = 60 min (± 30 min in their terminology), $Th_{\mathrm{A}}$ = 3 TECU/h and $Th_{\mathrm{R}}$ = 75%.

**Figure 3:**
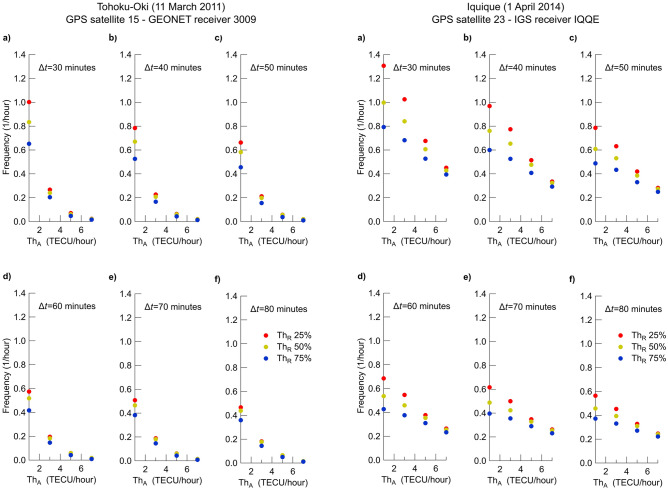
Positive breaks frequency detected in vTEC data versus absolute threshold ($Th_{\mathrm{A}}$), relative threshold ($Th_{\mathrm{R}}$, different colors), fitting window size $\Delta t$ (panels from a to f). Left side: 2011 (Tohoku-Oki earthquake). Right side: 2014 (Iquique earthquake).

Comparing our Fig. 2 to Heki and Enomoto’s (2015) Fig. 6 we observe a perfect match: we find exactly the same breaks and at the same times, thus supporting the validity of our own AIC algorithm. Supplementary information contains results from further validation: supplementary Fig. S1 reproduces quite well Fig. 3(a-1) by Heki and Enomoto^27^, supplementary Fig. S2 replicates Heki and Enomoto’s (2015) Fig. S6. As a test, in Supplementary Fig. S2 we display vTEC calculated considering all the elevation angles of the satellite. Once again, our algorithm is able to reconstruct all the breaks identified in Heki and Enomoto’s^27^ Fig. S6. Actually, our Supplementary Fig. S2 shows additional breaks outside the time interval they considered, at the edges of vTEC curves, when the GPS satellite has low elevation angles. These breaks are very likely related to multipath effects that become important when vTEC is calculated using signals from satellites that are low on the horizon. With regard to this issue, it’s worth highlighting that after comparing our results obtained using different values of the elevation angle threshold to those obtained by Heki and Enomoto’s^27^, we found that the threshold to use to reproduce their breaks and to limit multipath effects resulting in flawed breaks was of 20$^\circ$. We tried also higher elevation angle thresholds (i.e., 30$^\circ$ and 45$^\circ$), but in these cases some of the breaks were no more detected. At the same time we point out that information on the elevation angle threshold is missing in Heki and Enomoto^27^.

## Results and discussion

The successful validation of our algorithm allows performing a stress test on AIC method. Open questions on AIC method, to which we attempt to answer, concern: 1) the compatibility of breaks frequency with alert issuing for incoming strong earthquakes, 2) the possibility to univocally set AIC method parameters and 3) the possibility to relate detected breaks to sources other than earthquakes.

For each of the four considered earthquakes, we compute a 1-year vTEC time series from two GPS receiver-satellite pairs. Each time series has undergone the analyses shown below. Due to the coherence and similarity of results, here we include only those obtained using vTEC estimated from GEONET receiver 3009 and satellite 15 for the Tohoku-Oki earthquake, and from IGS receiver IQQE and satellite 25 for Iquique event. Results for the remaining six time series are displayed in the supplementary information.

To begin, we investigate the dependence of positive breaks frequency as a function of $\Delta t$, $Th_{\mathrm{A}}$ and $Th_{\mathrm{R}}$ considering vTEC data covering the whole 2011 for Tohoku-Oki earthquake and the whole 2014 for Iquique earthquake. We take fitting windows with size $\Delta t$ = 30, 40, 50, 60, 70 and 80 min, $Th_{\mathrm{A}}$ = 1, 3, 5, and 7 TECU/h, and $Th_{\mathrm{R}}$ = 25, 50, and 75%. The choice of these values is guided from those used by Heki and Enomoto^27^, who used values of AIC parameters for the different earthquake case studies, without clarifying the criterion for such a choice for each parameter. On the contrary, we explore a wide range of values to include all the cases they investigated and go even beyond, by testing AIC method for 72 different terns of $\Delta t$, $Th_{\mathrm{A}}$ and $Th_{\mathrm{R}}$.

Figure 3 shows how the hourly frequency of positive breaks depends on $\Delta t$, $Th_{\mathrm{A}}$ and $Th_{\mathrm{R}}$ for Tohoku-Oki (left side) and Iquique (right side) earthquakes. In this figure the hourly frequency of positive breaks always decreases when one parameter among $\Delta t$, $Th_{\mathrm{A}}$ and $Th_{\mathrm{R}}$ increases. Increasing $Th_{\mathrm{A}}$ and $Th_{\mathrm{R}}$ we are requesting more marked V-shaped vTEC changes; when increasing $\Delta t$, oscillating trends that could be better fitted with a piecewise linear function over small windows could be, instead, better fitted by a linear function over large windows.

Observing both sides of Fig. 3, and moving from panel a) to panel f) therefore considering increasing $\Delta t$, we see the general decreasing trend of positive breaks frequency. This behavior holds also looking at a single panel and focusing on how frequency depends on either $Th_{\mathrm{A}}$ or $Th_{\mathrm{R}}$. Let now focus on the left side of Fig. 3 and set $Th_{\mathrm{A}}$ = 3 TECU/h (as done by Heki and Enomoto^27^ for their Fig. 6). For this value of $Th_{\mathrm{A}}$, the minimum value of positive breaks frequency is around 0.14 h$^{-1}$ (for $\Delta t$ = 80 min and $Th_{\mathrm{R}}$ = 75%), while the maximum is around 0.27 h$^{-1}$ (for $\Delta t$ = 30 min and $Th_{\mathrm{R}}$ = 25%). Therefore, for $Th_{\mathrm{A}}$ = 3 TECU/h, the minimum break frequency is of one every $\sim$ 7 h, while the maximum is of one every $\sim$ 4 h. For $Th_{\mathrm{A}}$ = 1 TECU/h, break frequency increases up to about 1 per hour. Note that a value of $Th_{\mathrm{A}}$ = 1 TECU/h is set in the AIC analysis reported by He and Heki^2^ for three case studies of Mw $\ge$ 8.2 Chilean earthquakes in which they claim to have identified pre-earthquake TEC anomalies, both positive and negative. A frequency of around about one break per day (i.e. 0.05 h$^{-1}$) is obtained for $Th_{\mathrm{A}}$ $\sim$ 5 TECU/h. However, for $Th_{\mathrm{A}}$ 5 TECU/h the method loses sensitivity. The break identified by Heki and Enomoto^27^ before Tohoku-Oki earthquake disappears in our analysis for $Th_{\mathrm{A}}$ > 3 TECU/h.

Right side of Fig. 3 displays that even larger values of the maximum and minimum frequencies are obtained in the case of Iquique earthquake. Again setting $Th_{\mathrm{A}}$ = 3 TECU/h, we find that the minimum value of positive breaks frequency is around 0.28 h$^{-1}$, while the maximum is around 0.80 h$^{-1}$. The resulting minimum break frequency is of one every $\sim$ 4 h, while the maximum is of one every $\sim$ 1 h.

Supplementary Figs. S3 and S4 are equivalent to Fig. 3 but obtained using vTEC data for the two North Sumatra (main shock and aftershock) earthquakes, respectively, while supplementary Fig. S5 shows results for the remaining two vTEC time series for the Tohoku-Oki and Iquique earthquakes. Results shown in these supplementary figures very well agree with those of Fig. 3.

**Figure 4:**
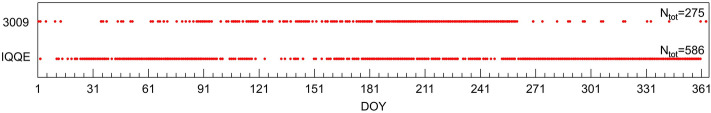
Yearly positive breaks. Positive breaks detected in vTEC derived from GPS satellite 15 and GEONET receiver 3009 (top) and GPS satellite 23 and IGS receiver IQQE (bottom) plotted as a function of the day of year (DOY). The total number of breaks (Ntot) found by AIC method (Δ*t* = 60 min, *Th*_A_ = 3 TECU/h^−1^ and *Th*_R_ = 75%) is written on the right side of the plot.

Figure 4 displays the distribution of the breaks found by AIC method along the days of the year (DOY) for the two cases of the Tohoku-Oki and Iquique earthquakes above discussed. Supplementary Figure S6 displays, analogously to Fig. 4, the distribution of the breaks found by AIC method for all the eight time series.

Our analysis shows that relevant positive breaks are observed with a frequency by far greater than that obtained by Heki and Enomoto^27^, i.e. 0.05 h$^{-1}$ and than that, i.e. 0.01 h$^{-1}$, they suggest as a threshold for considering pre-earthquake breaks as random.

Once assessed the way breaks frequency depends on the values of the adjustable parameters, next would be to tune them, if possible, to reduce AIC method’s sensitivity decreasing the breaks frequency to a reasonable value, without losing those breaks that are claimed to be precursory to strong earthquakes. We therefore record, for the eight analyzed vTEC time series, the terns (among the 72 we analyzed) for which the break observed prior to the earthquake is visible. We find that in most cases for $Th_{\mathrm{A}}$ $\ge$ 3 TECU/h the break that, according to Heki and Enomoto^27^ should be the precursor of the coming earthquake, vanishes. This means that when raising the thresholds to reduce frequency, in most cases AIC method is no more able to pick the breaks proposed as precursors. Differently, in the case of Iquique the break that Heki and Enomoto^27^ indicate as the precursor of the event is always present, independently of the tern used. However, this cannot be considered as evidence to support the seismogenic origins of the Iquique break, an interpretation of this finding is given in the following.

**Figure 5:**
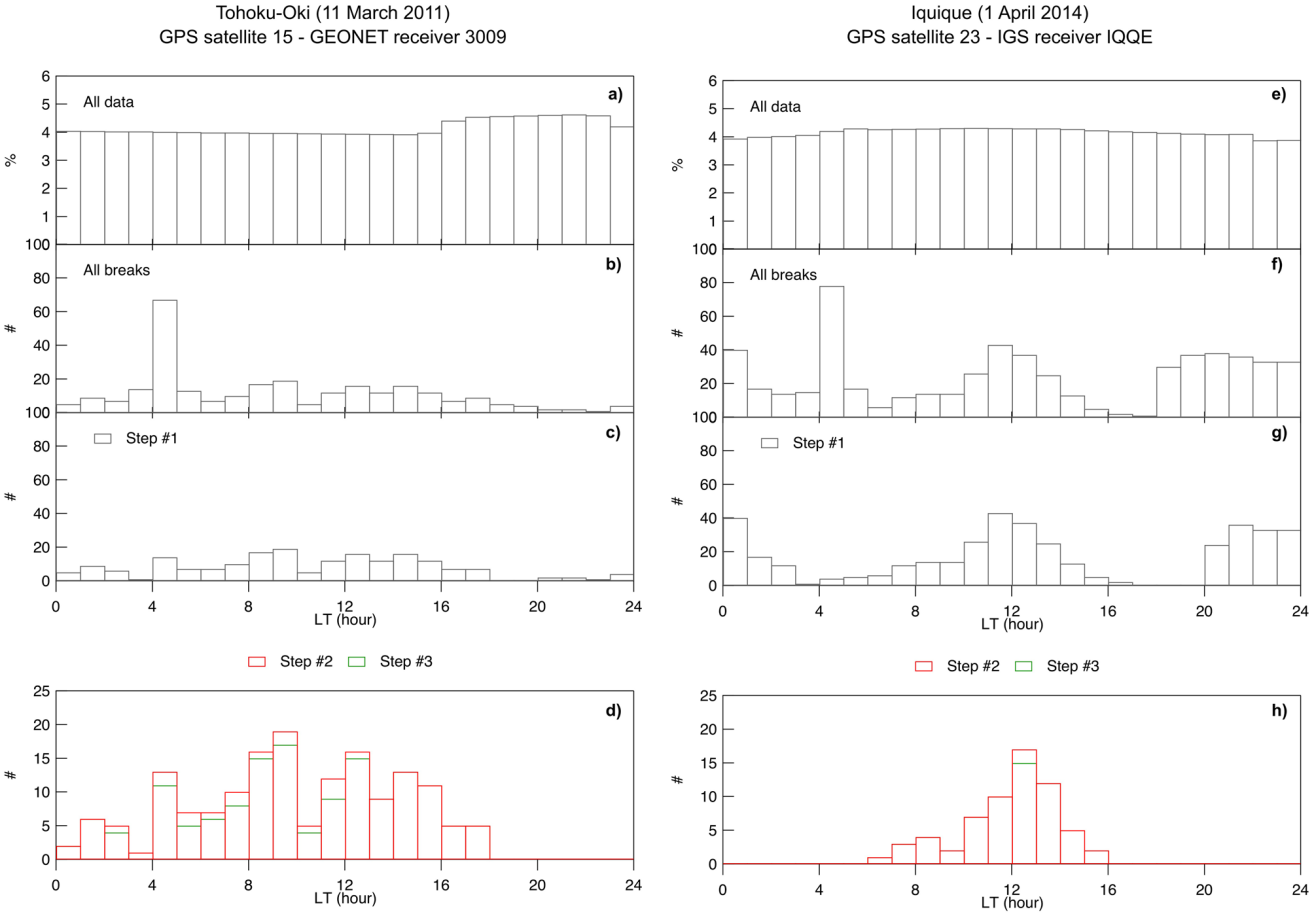
PDF of the LTs of positive breaks found by AIC method. From top to bottom, PDF of: (**a**,**e**) original vTEC values; (**b**,**f**) positive breaks found by AIC method; (**c**,**g**) positive breaks after Step #1. The lowermost panels d and h display the PDFs of the positive breaks after Step #2 (red) and Step #3 (green). Left side shows results for Tohoku-Oki earthquake, right side results for Iquique earthquake.

To investigate the relation of positive breaks to non-lithospheric physical processes, we study the possible dependence of breaks occurrence on local time, solar and geomagnetic activity. To unveil this point, we follow an approach based on three steps, starting from the investigation of the dependence of the occurrence of positive breaks on local time (LT). For each break, we calculate its local time and estimate the related probability density function (PDF). Results are shown in Fig. 5 for Tohoku-Oki (left side) and Iquique (right side) earthquakes. In both cases we use $\Delta t$ = 60 min, $Th_{\mathrm{A}}$ = 3 TECU/h and $Th_{\mathrm{R}}$ = 75%. The top panels, a) and e), show the PDF of the LTs corresponding to the single values of analyzed vTEC to show that no initial bias in the LT distribution of vTEC data is present.

Figure 5b,f display the PDF of the LTs of positive breaks found by AIC method. The first feature emerging from both Fig. 5b,f is the large percentage of breaks occurring in the early morning; around 5:00 LT we observe a sort of clustering of breaks that could be ascribed to the steep electron density gradients typical of solar terminator hours^30–32^. To test this hypothesis we estimate the LT of the solar terminators (sunrise and sunset) at the time of each break. The first step (Step #1) in the identification of breaks due to Sun-Earth relation processes, consists of removing those breaks occurring at a LT falling within 1 h from that of sunrise and sunset; Fig. 5c,g show the resulting PDFs. Most of the breaks that were present in Fig. 5b,f around 5:00 LT have now disappeared thus supporting the hypothesis that part of the breaks found by AIC method in vTEC are ascribable to processes occurring at solar terminators.

**Figure 6:**
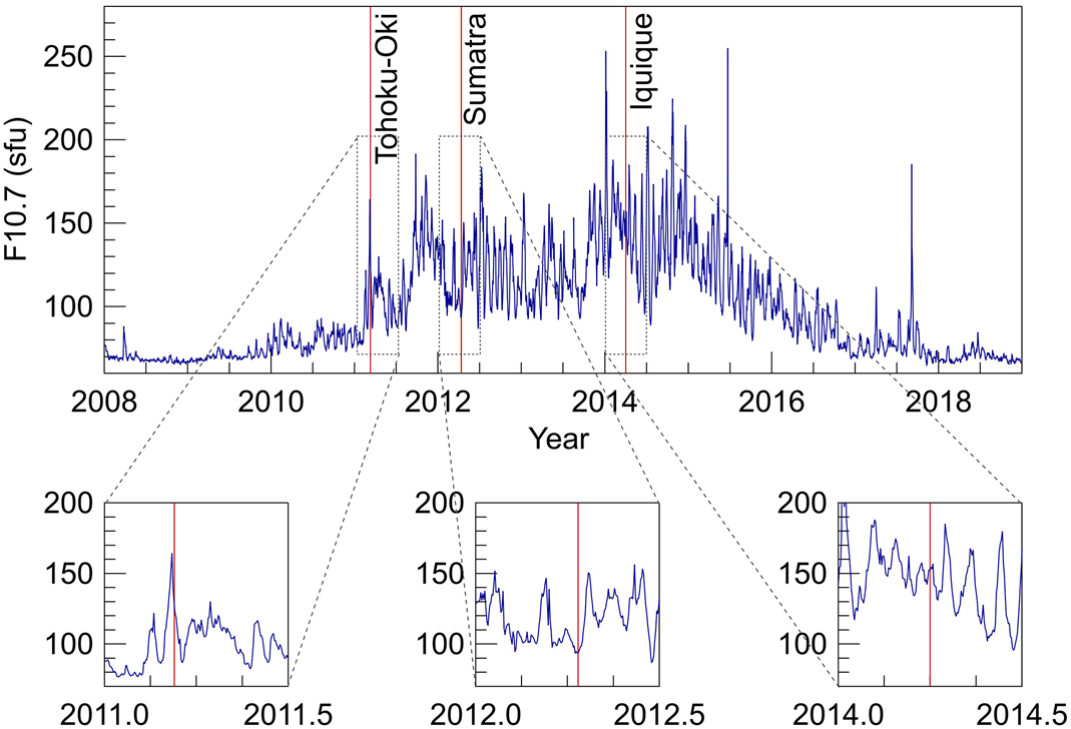
Solar activity represented by the daily F10.7 index (blue). Vertical red lines represent the times of the considered earthquakes. The enlarged views allow better showing where the considered events fall.

In Fig. 5f, and to a lesser extent also in Fig. 5b, the number of breaks increases around 12 LT. This trend could be again explained in terms of the variability of the ionosphere that usually reaches its maximum electron density value around noon^33^. Figure 5 evidences the interesting difference between the LT distribution obtained using data recorded in 2011 (Fig. 5b) and data recorded in 2014 (Fig. 5f). This difference can be explained in terms of the different level of solar activity for those years, and hence of the much higher rate of ionization during 2014 than during 2011. As visible in Fig. 6 displaying the daily F10.7 index^34^, in 2011 the solar cycle was just beginning, it reached its maximum in mid 2014. This further supports the hypothesis that part of the positive breaks that AIC method detects are due to both the regular (daily) and irregular variations vTEC undergoes because of the different exposure of different parts of the ionosphere to the Sun and by the different levels of solar activity.

In the second step (Step #2), we consider the daily F10.7 index to investigate whether some of the breaks are due to solar activity. In Step #2, we eliminate from the set of breaks shown in Fig. 5c,g those occurred for F10.7 > 120 sfu, namely under moderate-high solar activity. The updated PDFs are shown in red in Fig. 5d,h and fully support the aforementioned hypotheses.

To complete the picture of a possible dependence on the solar and geomagnetic activity we consider also the global geomagnetic index ap, the linearized version of the Kp index with a time resolution of 3 h. In the third step (Step #3), we eliminate from the remaining breaks those occurring when ap > 22, corresponding to moderate-high geomagnetic activity. The updated PDFs are displayed in green in Fig. 5d,h showing a further, slight, removal of breaks.

What happened to the breaks claimed by Heki and Enomoto^27^ as precursors of Tohoku-Oki and Iquique earthquakes? Both disappear, occurring respectively when F10.7 = 120.5 sfu and ap = 56 and when F10.7 = 153.1 sfu and ap =18. The analyses illustrated above are repeated also on the remaining six vTEC time series and shown in Figs. S7, S8 and S9 of the supplementary information.

Table 3 lists, for all the analyzed vTEC time series, the number of breaks removed in each step and the overall percentage of breaks that could be explained as a consequence of the influence on the ionosphere of the Sun or of space weather events. According to Table 3, out of the breaks identified by AIC method, a percentage ranging from 46 to 90% can be ascribed to the influence on the ionosphere of the Sun–Earth interaction. The high percentage of “explained” breaks found for the Iquique earthquake suggests that the discrimination of breaks due to non-solar sources is further complicated for years of high solar activity.

**Table 3:** Number of breaks ascribable to sources other than earthquakes. From left to right: total number of breaks detected by AIC method ($\Delta t$ = 60 min, $Th_{\mathrm{A}}$ = 3 TECU/h, $Th_{\mathrm{R}}$ = 75%), number of breaks removed after each of the three steps, the overall percentage of ‘explained’ breaks.

Earthquake (receiver)	Total No. of breaks	No. of breaks removed by Step #1	No. of breaks removed by Step #2	No. of breaks removed by Step #3	Overall % of ‘explained’ breaks
Tohoku-Oki (0221)	270	74	30	19	46
Tohoku-Oki (3009)	275	84	29	16	47
Sumatra main shock (BNON)	346	145	98	3	71
Sumatra main shock (LHW2)	345	148	95	0	70
Sumatra largest aftershock (LEWK)	570	223	178	5	71
Sumatra largest aftershock (UMLH)	352	141	108	2	71
Iquique (AREQ)	771	255	414	4	87
Iquique (IQQE)	586	184	339	2	90

## Conclusions

Meticulous tests on published pre-earthquake ionospheric anomalies (see, e.g., Masci et al.^23^; Kamogawa and Kakinami^25^; Utada and Shimizu^26^) have given rise to a lively scientific debate^35^ on the possible observation of precursory signatures shortly before the shock. The purpose of our investigation is to check the validity and robustness of AIC method in the detection of rapid variations in the vTEC rate of change hypothesized to be precursors of Mw > 8 earthquakes^27^.

After having validated our AIC algorithm by accurately reproducing the results obtained by Heki and Enomoto^27^, we investigated the role of the adjustable parameters ($\Delta t$, $Th_{\mathrm{A}}$ and $Th_{\mathrm{R}}$) on AIC method outcomes, also quantifying breaks frequency. In all the analyzed cases we found that break frequency is too high to propose AIC method as a possible alert tool to identify earthquake-related breaks and that raising the thresholds to lower breaks frequency makes, in most cases, AIC method insensitive to the pre-earthquake breaks found by Heki and Enomoto^27^. To quantify the number of breaks of solar and geomagnetic origin we counted the number of breaks that are explainable in terms of purely ionospheric sources, i.e. those occurring close to the solar terminators, and during moderate and high solar and geomagnetic conditions.

Based on our results, detected breaks can be clearly related to sources other than earthquakes. Our results refute Heki and Enomoto’s^27^ conclusion that breaks due to sources other than seismic ones (e.g., solar and geomagnetic activity) are not frequent enough. It is worth highlighting that to reproduce the work by Heki and Enomoto^27^ we analyzed data related only to a single satellite per receiver. We are confident that considering all the satellites in view we would have found a number of breaks by far larger, as recently found by Ikuta et al.^36^. Ikuta et al.^36^ reproduced Heki and Enomoto’s^27^ work and, differently from us, they considered two-month vTEC time series’ reconstructed for all the GPS satellites in view from a given receiver and for only one combination of AIC parameters ($\Delta t$ = 60 min, $Th_{\mathrm{A}}$ = 3 TECU/h, $Th_{\mathrm{R}}$ = 75%).

Considering that according to He and Heki^37^ AIC method is not useful for practical alerts of Mw ≤ 8 earthquake, and that in the last 50 years 24 Mw $>$ 8.0 earthquakes occurred, of which 5 with Mw $\ge$ 8.5 and 2 with Mw $\ge$ 9 (*https* : //*earthquake*.*usgs*.*gov*/*earthquakes*/*search*/), the question is “Can a warning system based on vTEC breaks be diagnostic of impending large earthquakes and then effective for public safety?”. The answer resulting from this study is that the AIC method detects too many breaks especially during high solar activity years, showing the breaks’ dependence on solar-related sources (i.e. solar terminator, local time, solar activity). This indicates that most, if not all of them, are not related to earthquakes. So, identifying possible ionospheric pre-seismic signals is extremely challenging, because too many false alarms would make the method unusable.

## Supplementary information

Supplementary information.

## Data Availability

All vTEC data analyzed in this paper are available at the following link: https://data.mendeley.com/datasets/2s9rwgdsj6/2.
