# Supplementary material for: A stress test to evaluate the usefulness of Akaike information criterion in short-term earthquake prediction
Source: Sci Rep. 2020 Dec 3;10:21153. doi: 10.1038/s41598-020-77834-0 (PMC7713355; doi:10.1038/s41598-020-77834-0)
Supplement: Supplementary file 1 — Supplementary information. [file 41598_2020_77834_MOESM1_ESM.pdf]

Supplemetary Information for "A stress test to evaluate the usefulness of Akaike information criterion in short-term earthquake prediction"

Roberta Tozzi[1,*], **Fabrizio Masci**[2], **and Michael Pezzopane**[1]

[1]Istituto Nazionale di Geofisica e Vulcanologia, Rome, 00143, Italy
[2]Istituto Nazionale di Geofisica e Vulcanologia, L'Aquila, 67100, Italy
[*]roberta.tozzi@ingv.it

ABSTRACT

This supplementary information contains texts and figures that enrich the main paper. In detail, supplementary discussion S1 reports some comments on the Heki and Enomoto's[1] attempt to reply to the issues raised by Masci et al.[2], while supplementary discussion S2 provides some details on the method used to derive equivalent vTEC from GPS data. Figures S1 and S2 show results from further validation made in addition to that displayed in the main text. Figures S3, S4 and S5 display the dependence of breaks frequency on AIC adjustable parameters (Δt, Th_{A} and Th_{R}) for the cases not included in the main text. Figure S6 illustrates breaks distribution for all the analyzed time series along the specific year. Figures S7, S8 and S9 display the PDF of the local times (LT) for the original vTEC time series, for AIC method detected breaks, after the three steps of breaks removal to check their origin due to the Sun-Earth interaction for the cases not included in the main text.

Supplementary Discussion S1: Can Heki and Enomoto (2015) be considered as a direct rebuttal to Masci et al. (2015)?

In italic, we report exactly what maintained by Heki and Enomoto[1].

Short summary of the debate

Heki[3] claimed that an anomalous increase of ionospheric sTEC started 40 minutes prior to the Mw 9.0 Tohoku-Oki earthquake on 11 March 2011, as well as before other large earthquakes, maintaining that the sTEC increase was likely earthquake-related. Heki and Enomoto[4] reached the same conclusions of Heki[3] by analyzing vTEC data instead of sTEC. In the two papers, the pre-earthquake TEC increase was identified as the deviation from a reference curve used as the normal TEC background. The reference curve was obtained by fitting the TEC time series before and after the earthquake time.

Masci et al.[2] addressed this reference curve method and demonstrated that the pre-earthquake sTEC increase reported by Heki[3] is not anomalous, but it should be regarded as an artefact rather than a precursor of the incoming earthquake. This because, the reference curve is obtained by the authors fitting also post-seismic sTEC data that are strongly disturbed by atmospheric pressure waves triggered by the earthquake that propagate upward, reach the ionosphere a few minutes after the shock perturbing it for hours.

More recently, Heki and Enomoto[1] suggested a new numerical approach based on the Akaike's information criterion (AIC), in order to identify pre-earthquake increases in the rate of TEC change (positive breaks) that they associate to the onset time of earthquake-related disturbances.

In the main paper, we have addressed the Heki and Enomoto's[1] results. Since Heki and Enomoto[1] argue that their paper is basically written as the direct rebuttal to Masci et al.[2], whose criticisms seem were just based on their misunderstandings, here we report some comment on the Heki and Enomoto's[1] attempt to reply to the Masci et al.[2] criticisms. Below we address all the criticisms following the Heki and Enomoto's[1] numeration.

Criticism #1: the reference curve method

Criticism #1 concerns the remark by Kamogawa and Kakinami[5] and Masci et al.[2] on the ambiguity of the reference curve method used both by Heki[3] and Heki and Enomoto[4] for identifying a pre-earthquake increase in TEC time series.

Heki[3] and Heki and Enomoto[4] showed that an increase started 40 minutes prior to the occurrence of Mw > 8 earthquakes in sTEC and vTEC data, respectively. The striking similarity of TEC pre-earthquake increases led the authors to hypothesize a seismogenic origin.

It can be noted that, a few minutes after the earthquakes, sTEC and vTEC time series reported in the two papers mentioned above show evident co-seismic ionospheric disturbances (CIDs) lasting for hours (see Heki[3] Fig. 2; Heki and Enomoto[4] Figs. 2 and 3). CIDs are usually observed in TEC data to appear shortly after very large earthquakes as ionosphere's response to atmospheric pressure waves propagating upward created by the sudden piston-like motion of the ground/ocean surface[6,7].

According to Masci et al.[2], the pre-earthquake TEC increase reported in Heki[3] and Heki and Enomoto[4] is identified by using a flawed reference curve as the normal TEC background. To be more precise, Masci et al.[2] put in evidence that:

i) In both papers, the normal TEC background is defined by a polynomial fitting of TEC time series excluding 1 hour period of data, from -40 to +20 minutes with respect to the earthquake time (see, e.g., Fig. 8 in Heki and Enomoto[4]).

ii) CID mainly results as a sudden depletion of the TEC lasting for hours for large earthquakes, to which resonant atmospheric oscillations are superimposed. Kakinami et al.[8] named "ionospheric hole" the sudden long-lasting post-seismic TEC depletion. Astafyeva et al.[9] interpreted the TEC hole as the negative phase on an N-like shape wave, a compression-rarefaction wave generated by the earthquake.

iii) The amplitude and duration of a CID mainly depend on the earthquake magnitude. That is, stronger earthquakes cause larger and longer TEC perturbations[9]. Usually, CIDs generated by large earthquakes are observed lasting from 1 to a few hours before TEC recovers the normal state.

iv) Considering the 2011 Tohoku-Oki earthquake case studies, sTEC and vTEC time series reported by Heki[3] and Heki and Enomoto[4] show the onset of a CID effect at about 10 minutes after the shock. This is followed by a significant post-seismic depletion (the hole) that lasts at least 2 hours after the shock, more than the 20 minutes period of data excluded in the Heki's fitting procedure. TEC curves also show resonant atmospheric oscillations superimposed to the depletion. Saito et al.[10] show that the area of the TEC depletion after the Tohoku-Oki earthquake had horizontal size of about 500 km.

Masci et al.[2] concluded that it is evident that such post-seismic long-lasting depletion strongly influences the definition of the reference curve. This because, Heki[3] and Heki and Enomoto[4] used in the fitting procedure TEC data with a deep post-seismic depletion. This generates an apparent pre-seismic TEC increase that results in the identification of a flawed pre-earthquake TEC increase.

We would like to put in evidence, that Heki and Enomoto[1], while trying to refute the Masci's criticism, at page 7007 state that:

- *The reference curve method is essentially impractical for short-term earthquake prediction. Even if we could constrain the onset of the anomalies, we need the TEC data after earthquakes to pin down the reference curves (extrapolation from the preseismic part is hardly satisfactory).*
- *As the response to Criticism #1, we will propose a new approach to identify "breaks" (abrupt increase in rate) in absolute vTEC time series as a substitute for the reference curves (section 2.3).*

Still at page 7008:

- *In both Figures 1 and S2, we could intuitively recognize preseismic vTEC increase and postseismic recovery. There, we drew "reference curves" as we did in Heki and Enomoto (2013). Although it became easier to identify the onset of the anomaly by the sTEC to vTEC conversion, we still need the data after earthquakes to draw such reference curves.*

Note that, Heki and Enomoto[1], in a very unusual way for a rebuttal, agree with Masci et al.[2] that the reference curve method is a flawed method for identifying pre-earthquake increases in TEC time-series because data after the earthquake time are required. Therefore, they explore a new method in order to identify anomalous pre-earthquake TEC changes.

In conclusion, we do not see in Heki and Enomoto[1] any rebuttal of the criticism #1.

Mw dependence and reference curve method

Heki[3] (see his Fig. 4b) shows a dependence between the size of the TEC anomaly and the earthquake magnitude Mw, that is, stronger earthquakes are preceded by larger pre-earthquake TEC increases.

Masci et al.[2] pointed out that the dependence of the anomaly size with Mw is seemingly induced by the reference curve method. To be more precise, Astafyeva et al.[9] have shown that the depth of the post-seismic TEC depletion mainly depends on the earthquake magnitude. That is, stronger earthquakes trigger deeper post-seismic TEC depletions. Therefore, by using the Heki's reference curve method, a deeper post-seismic TEC depletion results in the identification of a larger pre-earthquake TEC increase.

Note that at page 7010 Heki and Enomoto[1] state that Heki[3], while claiming a direct dependence between the earthquake magnitude and precursor size, *in its Fig. 4b, compared sizes of the precursors of four earthquakes as the cumulative departure of the data from the reference curves at their occurrence times. This quantity, however, depends on the definition of reference curves.*

Once again, unexpectedly for a rebuttal, Heki and Enomoto[1] agree with Masci's remark maintaining that the reference curve method identifies pre-earthquake increases whose amplitude in induced by analysis procedure and also leads to an apparent direct relationship between Mw and the pre-earthquake anomaly size.

Criticism #2: natural variability of TEC

As a further investigation of the TEC anomalous pre-earthquake increase shown in Heki[3] before the 2011 Tohoku-Oki earthquake, Masci et al.[2] performed a superposed epoch analysis of sTEC curves ($\pm$ 30 days around the earthquake time). They show that the TEC increase reported by Heki[3] was not particularly anomalous, but it may be explained in terms of normal global-scale ionospheric variability.

Concerning to this criticism, Heki and Enomoto[1] maintain that:

- page 7007: *Rebuttal to Criticism #2 is not straightforward because we agree that the natural variability overwhelms the precursors in terms of amplitudes especially when geomagnetic activity is high.*
- page 7018: *We did not simply rebut to Criticism #2.*

Thus, Heki and Enomoto[1] agree with Masci et al.[2] rather than refute their criticism.

Criticism #3: the simultaneous $\sim$ 40 minutes onset of TEC anomalies prior to different earthquakes

Heki and Enomoto[1] (page 7007) claim that Masci et al.[2] *considered it unnatural and wrong that all the reported pre-seismic electron enhancement started $\sim$ 40 min before earthquakes in spite of the diversity in earthquake magnitudes and mechanisms. Criticisms #3 seem to come from misunderstandings by Masci et al. (2015). In section 3.3, we show that the onset time varies from 80 min (2004 Sumatra-Andaman) to 25 min (2014 Iquique) before earthquakes, and they depend on Mw and earthquake types.*

Heki[3] and Heki and Enomoto[4] showed that anomalous, yet similar, increases of TEC started 40 minutes prior to the 2011 Tohoku-Oki, as well as before other Mw>8 earthquakes. Masci et al.[2] strongly stressed that if the 40 minutes exceptional repeatability had seismic origin, it should be related to a geological and tectonic context that must be necessarily common to the areas where the earthquakes occurred. That is, strong earthquakes occurred in different regions of the Earth were able to activate a mechanism that generated pre-earthquake TEC anomalies at the same lead-time of 40 minutes. However, this cannot be considered a realistic scenario because the earthquakes investigated by Heki (2011) and Heki and Enomoto[4] had very inhomogeneous seismological properties, and were generated in completely different tectonic and geodynamic scenarios.

Anyway, while not considering the geological context, by excluding TEC data from -40 to +20 minutes with respect to the earthquake time in defining the reference curve, is evident that Heki[3] and Heki and Enomoto[4] *a priori* considered TEC data outside this range as the normal TEC background. Consequently, they a-priori make a decision regarding the onset time, the observation period, and the amplitude of the pre-earthquake TEC anomaly they are looking for.

Note also that Heki and Enomoto[1] by using the AIC method for the same cases studies of Heki[3] and Heki and Enomoto[4], where the curve fit method is used, identified different onset times for pre-earthquake TEC increases. For instance, for the 2004 Sumatra-Andaman earthquake, the onset time changes from -40 to -80 minutes, while, for the 2014 Iquique it goes from -40 to -25 minutes. Only for Tohoku-Oki earthquake, Heki and Enomoto[1] confirmed the break detection 40 minutes prior to the shock.

In summary, in Heki and Enomoto[1] there is no rebuttal to the Masci's criticism #3. Instead, it is evident that the Heki and Enomoto's[1] AIC analysis does not support the 40 minutes repeatability of the onset of pre-earthquake anomalies claimed by Heki[3] and Heki and Enomoto[4].

Criticism #4: the anomaly in the magnetic declination at the time of the 2011 Tohoku earthquake

Heki and Enomoto[4] (Figs. 4 and A3) compared magnetic data of Kakioka and Kanoya station at the time of the Tohoku-Oki earthquake. They show a pre-earthquake anomaly in the magnetic declination D at Kakioka. The D anomaly seems to arise almost simultaneously with the TEC anomaly 40 minutes before the earthquake. No anomalies seem to be presents in components H and Z, and in the total field F. According to them, this suggests that the earthquake-related perturbing magnetic field is dominantly in east-west direction.

At page 7007 Heki and Enomoto[1] state that Masci et al.[2] *commented on the geomagnetic field and thought it unlikely that the pre-seismic anomaly $\sim$ 40 min before the Tohoku-Oki earthquake is seen only in the declination time series (Criticism #4). They also maintain on that this criticism seem to come from misunderstandings by Masci et al. (2015).*

The main remark by Masci et al.[2] concerned the idea that the perturbing magnetic field was dominantly (i.e., almost polarized) in the east-west direction. According to Masci et al.[2], it is hard to accept that:

i) The existence of an earthquake-related effect able to generate polarized magnetic disturbances.

ii) The idea that alleged earthquake-related polarized disturbances may preserve polarization before being observed.

Finally, Masci et al.[2] (Fig. 5) put in evidence that Heki and Enomoto[4] identified the D anomaly by using a curve fit method similar to that they used for the TEC anomaly. Therefore, it is very likely that also the D anomaly should be an artifact due to the fitting procedure, where, as for TEC, both the onset and the end time of the anomaly are imposed by the authors.

Note that, Heki and Enomoto[1] at page 7018 agree with the Masci's conclusion stating that: *It is true that only declination showed the "clear" changes with the reference curve method.* Thus, once again, we do not see any rebuttal of the criticism #4.

A further Heki's remark on Masci et al. (2015): sTEC or vTEC?

Heki and Enomoto[1] (page 7006) criticized the choice of Masci et al.[2] *which analyze sTEC time series stating that they did not give a reason why they did not use absolute vTEC, but use sTEC.*

According to Heki and Enomoto[4], vTEC time series allow a better identification of pre-earthquake anomalies because are free from TEC U-shaped long-term changes seen in sTEC. We would like to point out that Masci et al.[2] after testing the results by Heki[3] did not consider necessary to replicate their analysis using vTEC data. This because, by using the curve fit method, both Heki[3] and Heki and Enomoto[4] show the same flawed 40 minutes repeatability for the onset time of pre-earthquake TEC increases.

Note that Heki and Enomoto[1] at page 7008 state: *In both Figures 1 and S2, we could intuitively recognize preseismic VTEC increase and postseismic recovery. There, we drew "reference curves" as we did in Heki and Enomoto[4]. Although it became easier to identify the onset of the anomaly by the sTEC to vTEC conversion, we still need the data after earthquakes to draw such reference curves.*

Concerning this point, we would like to stress that is surprising that in Kelley et al.[11], a paper co-authored by Heki, the authors:

i) Analyzed sTEC and not vTEC data, which according to Heki and Enomoto (2013) are more suitable for identifying pre-earthquake TEC changes.

ii) In addition, concerning Criticism #1, they authors still use the (flawed) reference curve method despite that previously Heki and Enomoto[1] have hypothesized the AIC method is more efficient in the search of pre-earthquake increases in vTEC time series.

Therefore, we can conclude that the conversion from sTEC to vTEC cannot be considered a key point in the study of the occurrence of pre-earthquake increases in TEC time series, but this is just a reorganization of the same data. This because, both using sTEC and vTEC, to define the reference curve in the Heki's papers authors needs of post-seismic TEC data perturbed by a sudden long-lasting post-seismic depletion.

Conclusions

Here we addressed the alleged Heki and Enomoto[1] rebuttal to criticism by Masci et al.[2]. The aim of a rebuttal is to refute what has been reported in a previous published paper. However, we have shown that Heki and Enomoto[1], while suggesting a new numerical approach in the search of pre-earthquake anomalies in TEC data, cannot be actually considered a direct rebuttal of Masci et al.[2] criticisms on Heki[3] and Heki and Enomoto[1], neither Masci's criticisms can be considered to come from authors misunderstandings, as claimed by Heki and Enomoto[1].

Supplementary Discussion S2: Description and processing of GPS-TEC data

Receiver-independent exchange (RINEX) files containing GPS code and carrier phase observables acquired every 30 seconds are used to obtain calibrated TEC values by applying the Ciraolo et al.[12] method, improved several times in the years (details at $https://drive.google.com/file/d/1tDn0kqtJSZllJbrHmZC9TLiJUEb3bo8-/view?usp=sharing$). To avoid problems related to the use of leveled observations, this method tries to estimate the phase offsets β_{arc} affecting the observations of the differential phase delay of each arc, i.e. S_{arc}

$$S_{\mathrm{arc}} = \mathrm{sTEC}_{\mathrm{arc}} + \beta_{\mathrm{arc}} \tag{S1}$$

To accomplish this task, sTEC values are mapped as a two-dimensional (2D) surface by means of the classical thin shell method (at an altitude that is typically set to 350 km)

$$\mathrm{sTEC} = \mathrm{vTEC} \cdot \sec\chi \tag{S2}$$

where vTEC, which is the equivalent vertical TEC, is a 2D unknown function over the thin shell and χ the angle formed by the line of sight receiver-satellite and the perpendicular to the shell at the ionospheric pierce point. It is worth highlighting that

the fact that vTEC is uniquely defined only for observations from a single station limits the applicability of the method to a multi-day, single-station solution. vTEC is expanded as a polynomial, linear in local time and of the fourth-order in *Modip*, the modified dip latitude proposed by Rawer et al.[13]. In virtue of the aforementioned considerations, the observations S_{arc} can be expressed as

$$\mathrm{S}_{\mathrm{arc}} = \sec \Sigma_n c_n p_n(\mathrm{LT}, Modip) + \beta_{\mathrm{arc}}, \tag{S3}$$

where p_n is the term of the polynomial of order n and c_n the corresponding coefficient. Equation (S3) is linear in the unknown coefficients c_n and phase offsets β_{arc}, and so can be solved via standard or more sophisticated least squares methods.

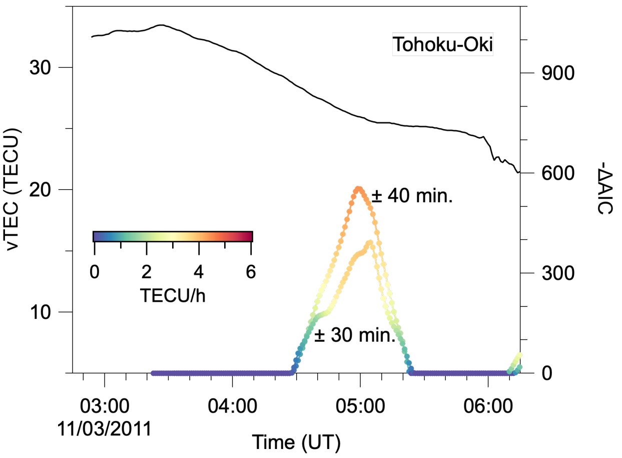

Figure S1. Our reproduction of Fig. 3 (a-1) by Heki and Enomoto[1]. We have analyzed the same GPS data from GEONET Satellite 15 and receiver 3009 by using the AIC algorithm we developed and the same AIC parameters, i.e., Δt = ±30 and ±40 min, Th_{R}=75%, Th_{A} =3 TECU/hour.

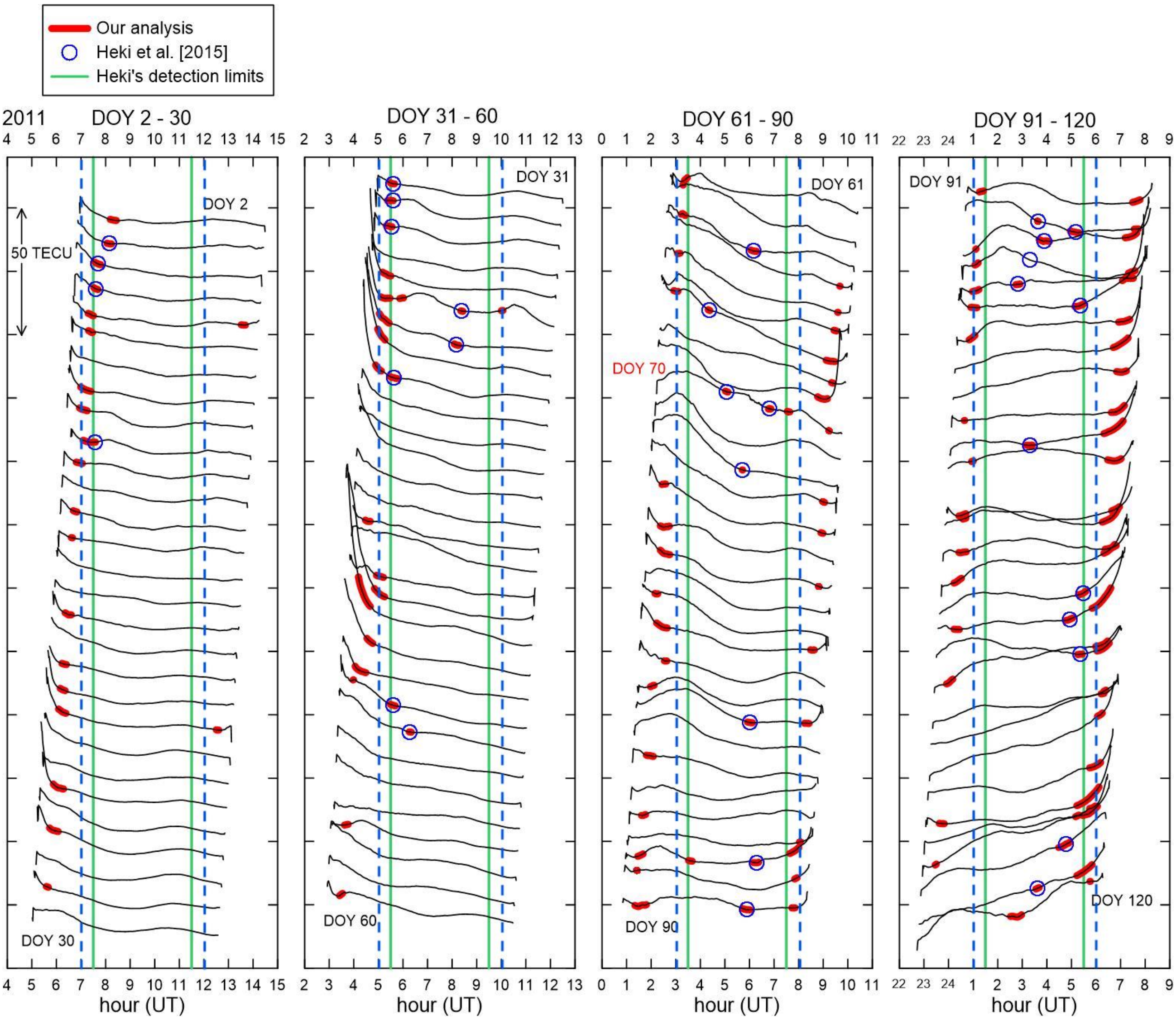

Figure S2. vTEC time series obtained from measurements made by the GEONET receiver 3009 and related to the GPS satellite 15, from January, 2 to April, 30 2011, considering all elevation angles. These data have been used to estimate AIC using $\Delta t = \pm 30$ minutes, Th_R=75%, Th_A =3.5 TECU/hour, i.e. the same parameters used by Heki and Enomoto[1], in order to replicate their Figure S6. The red thick segments identify the breaks we found, while the blue circles correspond to the breaks found by Heki and Enomoto[1]. Green vertical lines mark off the breaks detection limit of Heki and Enomoto[1]. Indeed, they seemingly looked for breaks in a time interval of 5 hours (delimited by the blue dashed vertical lines) but due to the 1-hour length of the AIC fitting window here used and to the way AIC method works, breaks can be actually found only in the time interval delimited by the green lines since it is not possible to estimate AIC values for the first and last 30 minutes of the considered time series. Note that we have found all the breaks identified by Heki and Enomoto[1] with the only exception of the one in DOY=94.

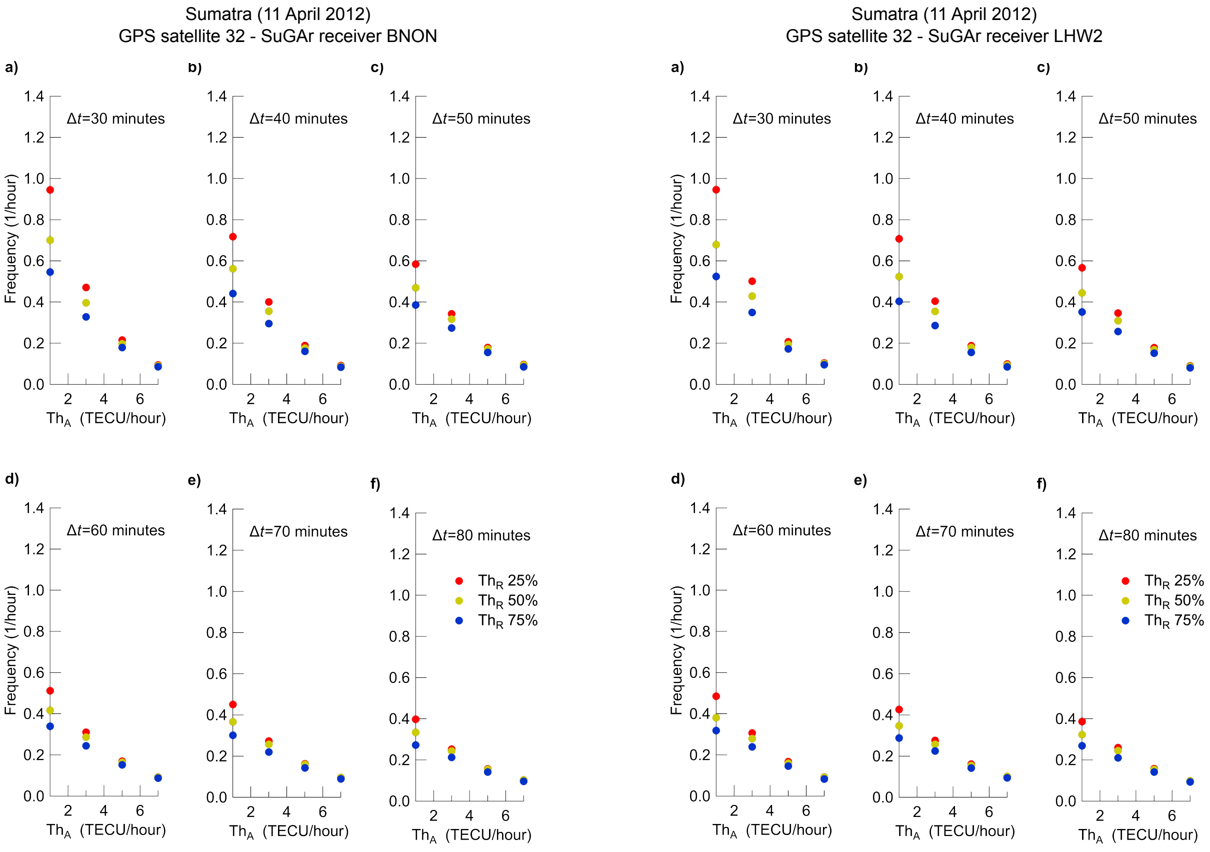

Figure S3. The same of Fig. 3 in the main text for 11 April 2012 North Sumatra main shock.

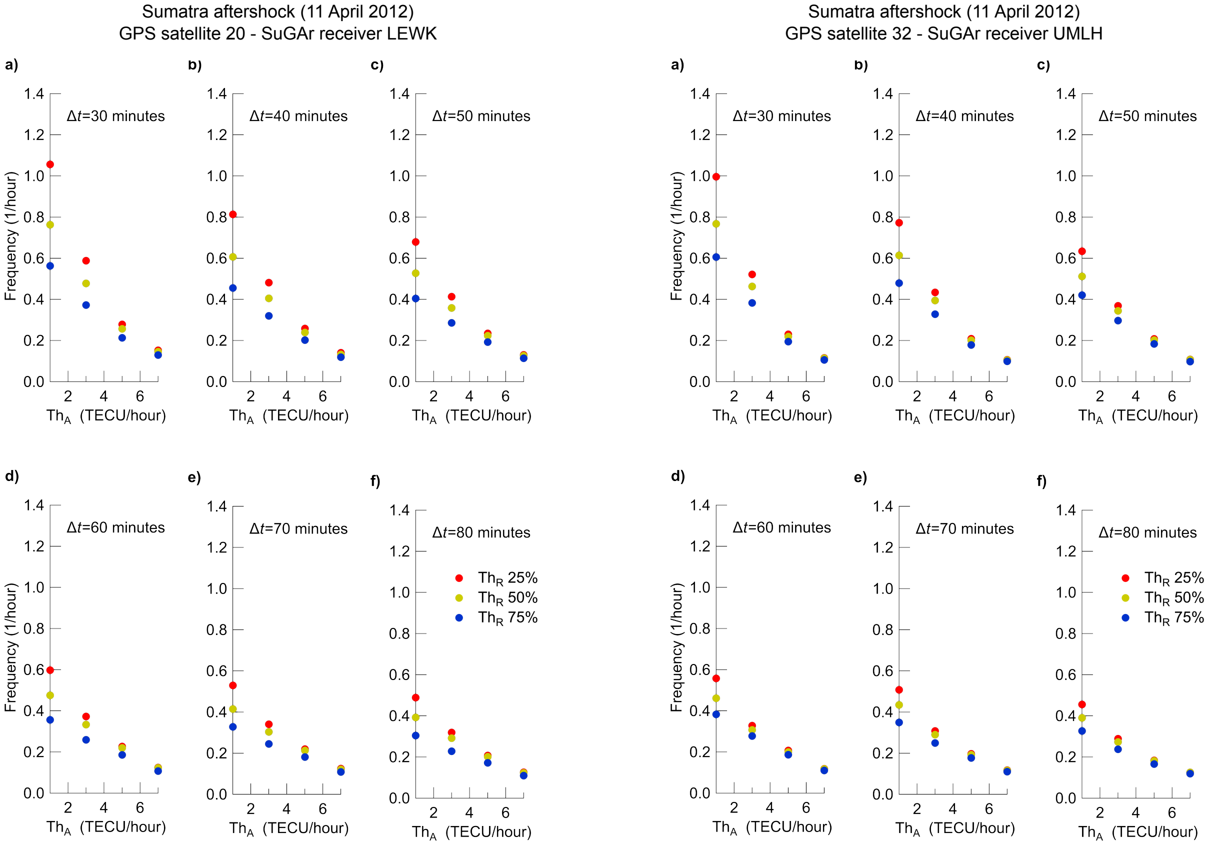

Figure S4. The same of Fig. 3 in the main text for 11 April 2012 North Sumatra largest after shock.

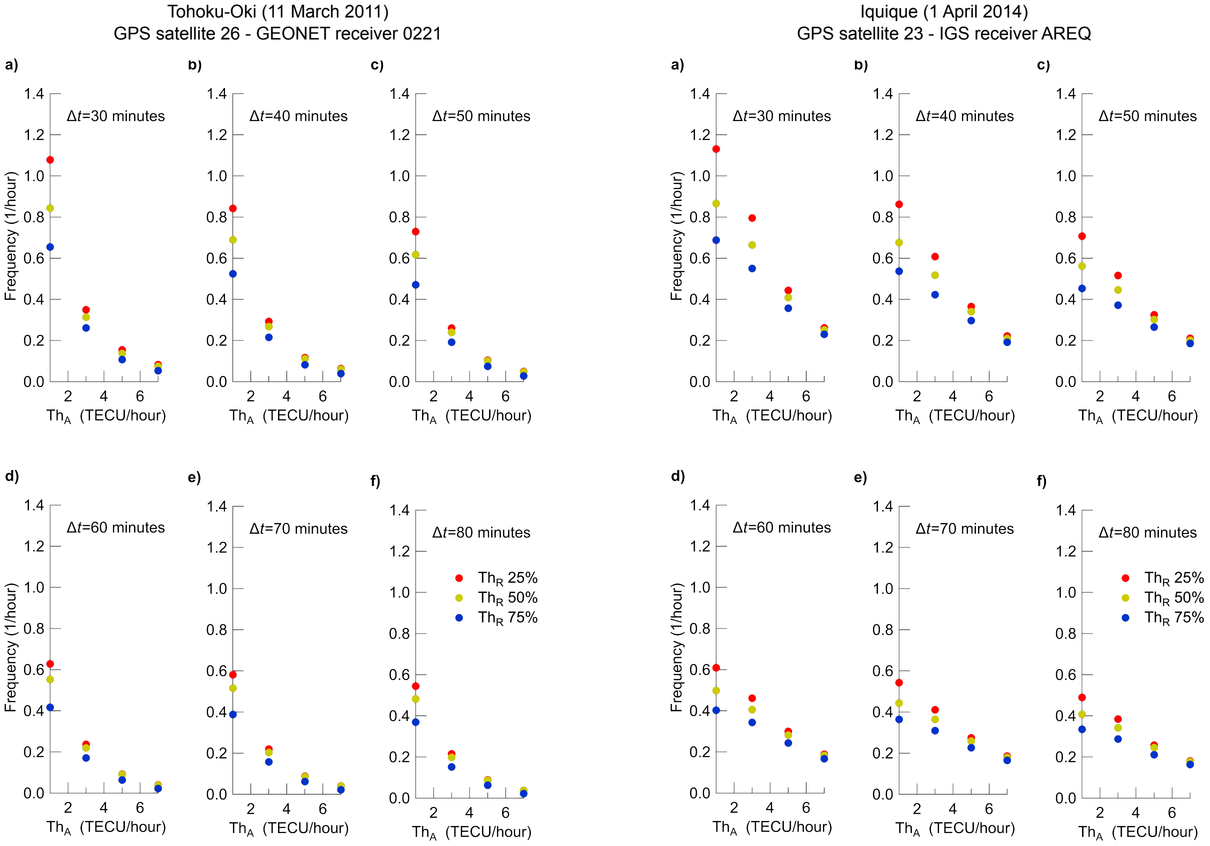

Figure S5. The same of Fig. 3 in the main text. Left side: Tohoku-Oki earthquake of 11 March 2011. Right side: Iquique earthquake of 1 April 2014.

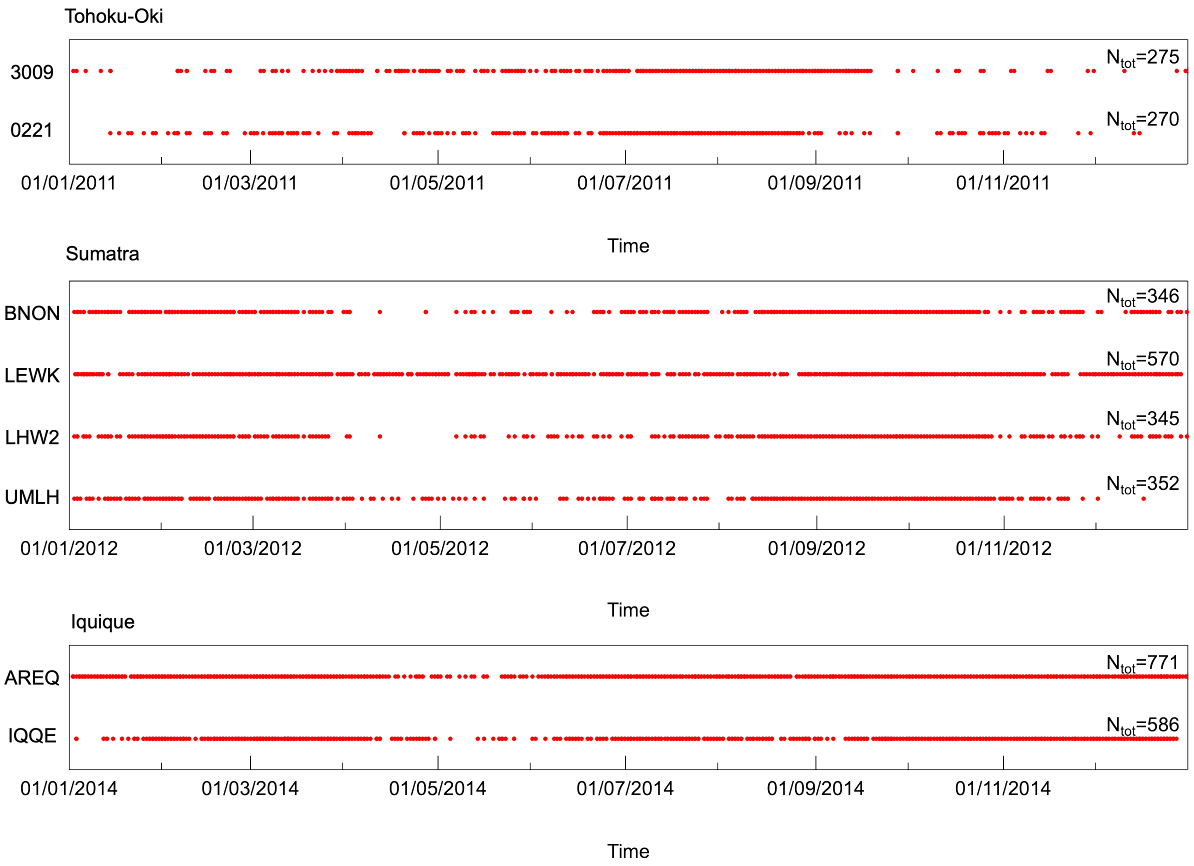

Figure S6. Positive breaks detected in vTEC derived from receiver-satellites pairs shown in Table 2 in the main text. Ntot indicates the total number of breaks found by AIC method (Δt = 60 minutes, Th_A=3 TECU/hour and Th_R=75%) in the specified vTEC time series.

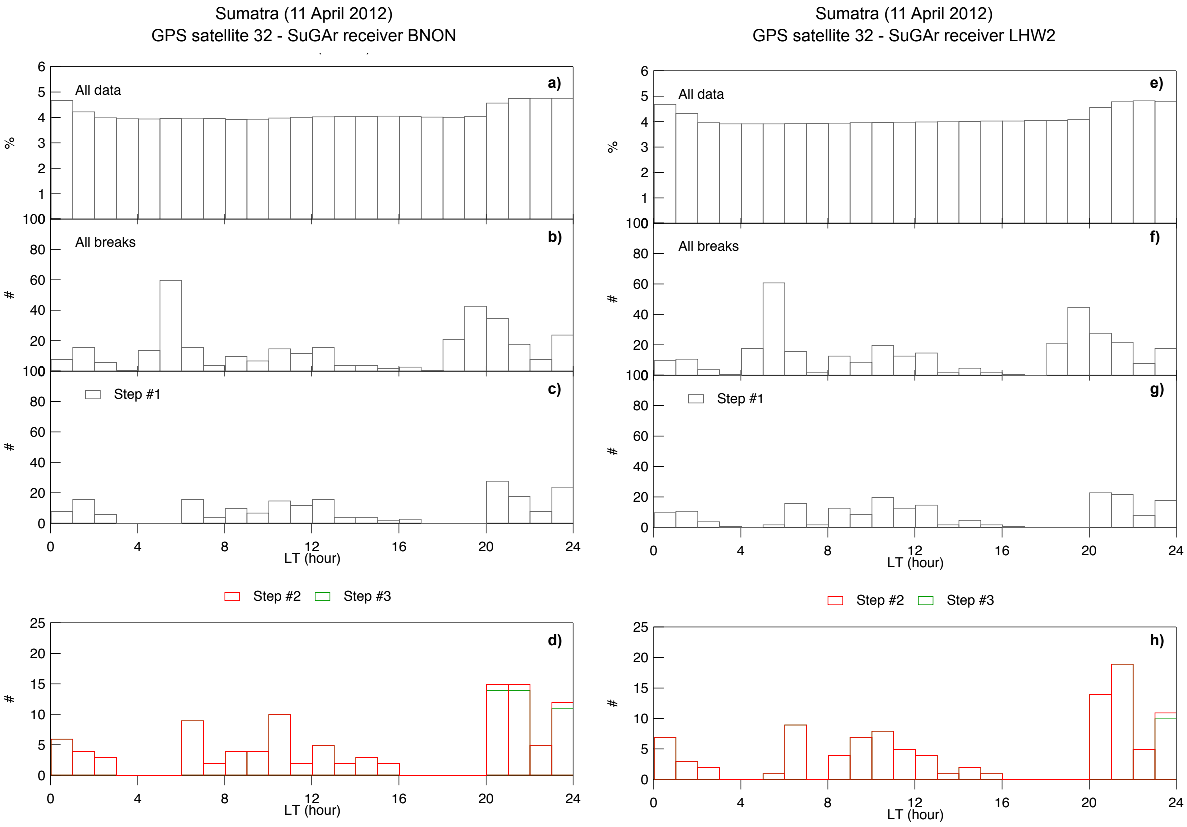

Figure S7. The same of Fig. 5 in the main text for 11 April 2012 North Sumatra main shock.

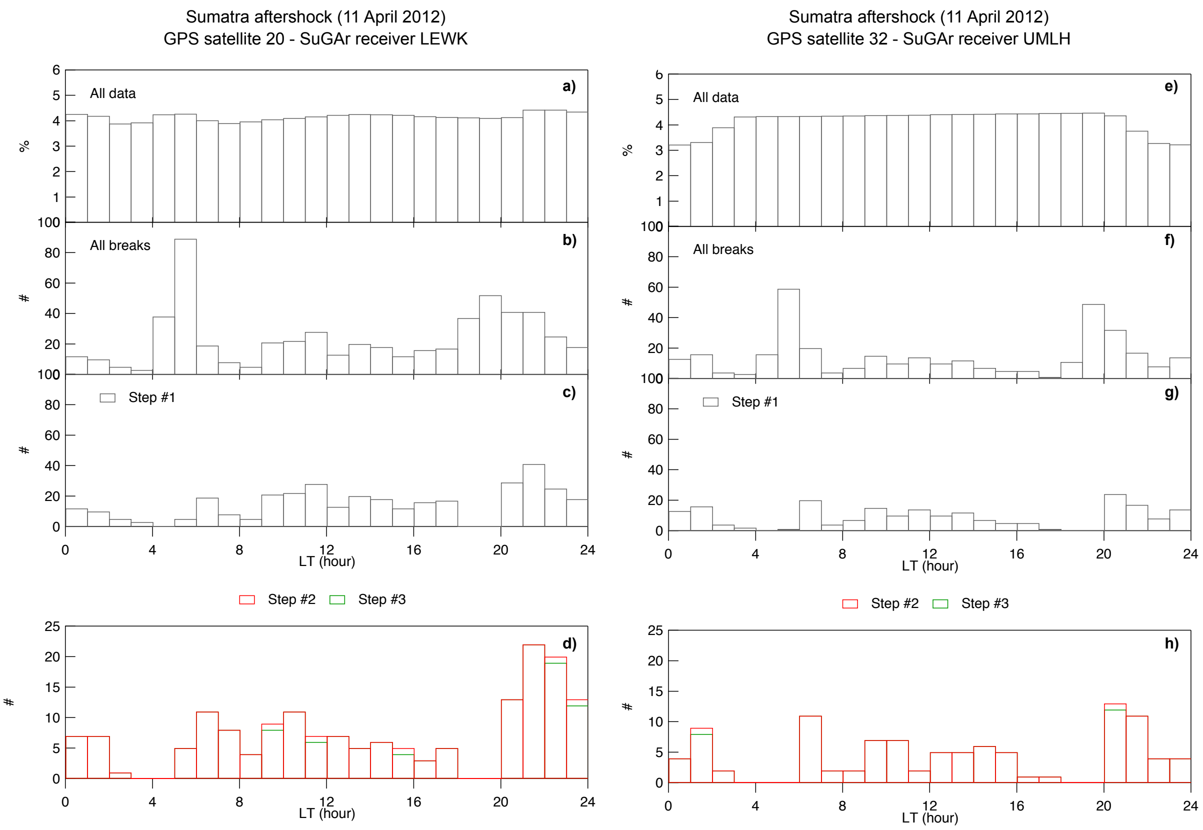

Figure S8. The same of Fig. 5 in the main text for 11 April 2012 North Sumatra largest after shock.

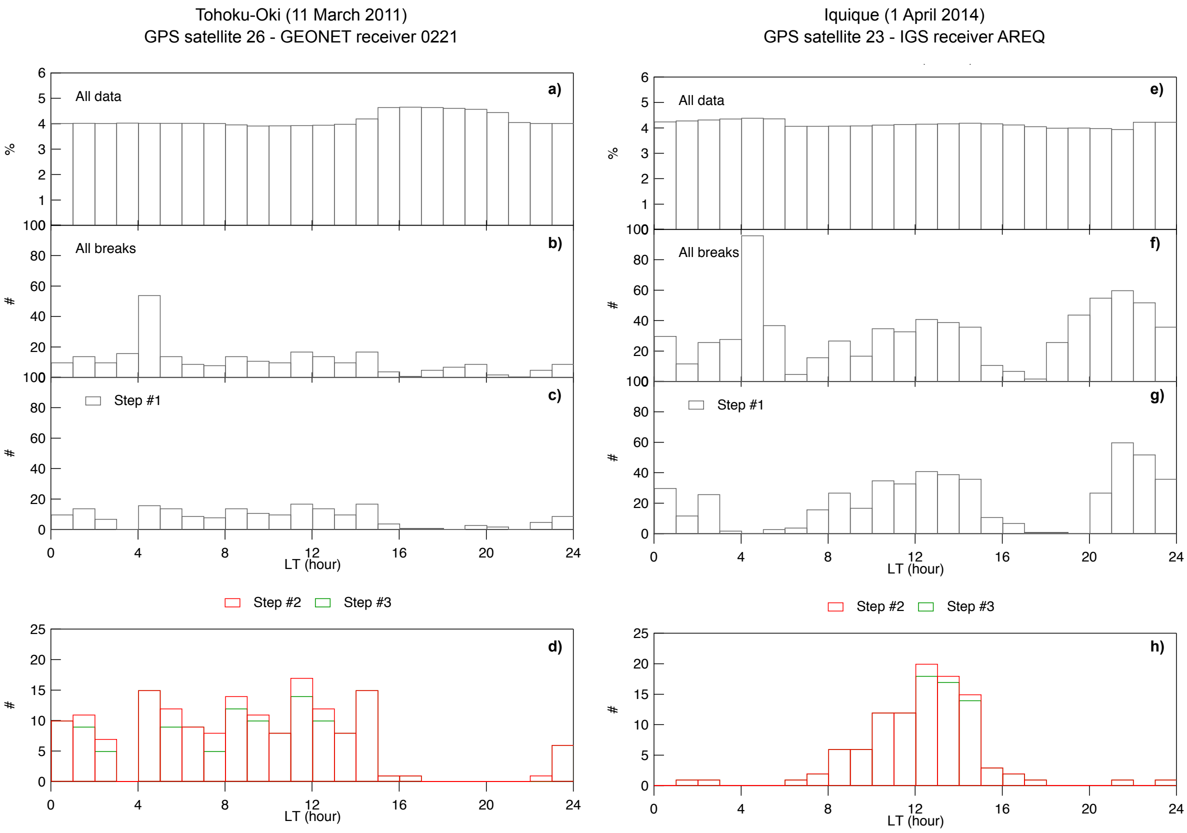

Figure S9. The same of Fig. 5 in the main text. Left side: Tohoku-Oki earthquake of 11 March 2011. Right side: Iquique earthquake of 1 April 2014.

References

1. Heki, K. & Enomoto, Y. Mw dependence of the preseismic ionospheric electron enhancements. *J. Geophys. Res. Space Phys.* **120**, 7006–7020, DOI: 10.1002/2015JA021353 (2015).
2. Masci, F., Thomas, J. N., Villani, F., Secan, J. A. & Rivera, N. On the onset of ionospheric precursors 40 min before strong earthquakes. *J. Geophys. Res. Space Phys.* **120**, 1383–1393, DOI: 10.1002/2014JA020822 (2015).
3. Heki, K. Ionospheric electron enhancement preceding the 2011 tohoku-oki earthquake. *Geophys. Res. Lett.* **38**, DOI: 10.1029/2011GL047908 (2011).
4. Heki, K. & Enomoto, Y. Preseismic ionospheric electron enhancements revisited. *J. Geophys. Res. Space Phys.* **118**, 6618–6626, DOI: 10.1002/jgra.50578 (2013).
5. Kamogawa, M. & Kakinami, Y. Is an ionospheric electron enhancement preceding the 2011 tohoku-oki earthquake a precursor? *J. Geophys. Res. Space Phys.* **118**, 1751–1754, DOI: 10.1002/jgra.50118 (2013).
6. Calais, E. & Minster, J. B. GPS detection of ionospheric perturbations following the January 17, 1994, Northridge Earthquake. *Geophys. Res. Lett.* **22**, 1045–1048, DOI: 10.1029/95GL00168 (1995).
7. Astafyeva, E. Ionospheric detection of natural hazards. *Rev. Geophys.* **57**, 1265–1288, DOI: 10.1029/2019RG000668 (2019).
8. Kakinami, Y. *et al.* Tsunamigenic ionospheric hole. *Geophys. Res. Lett.* **39**, DOI: 10.1029/2011GL050159 (2012).
9. Astafyeva, E., Shalimov, S., Olshanskaya, E. & Lognonné, P. Ionospheric response to earthquakes of different magnitudes: larger quakes perturb the ionosphere stronger and longer. *Geophys. Res. Lett.* **40**, 1675–1681, DOI: 10.1002/grl.50398 (2013).
10. Saito, A. *et al.* Acoustic resonance and plasma depletion detected by GPS total electron content observation after the 2011 off the Pacific coast of Tohoku Earthquake. *Earth, Planets Space* **63**, 64, DOI: 10.5047/eps.2011.06.034 (2011).
11. Kelley, M. C., Swartz, W. E. & Heki, K. Apparent ionospheric total electron content variations prior to major earthquakes due to electric fields created by tectonic stresses. *J. Geophys. Res. Space Phys.* **122**, 6689–6695, DOI: 10.1002/2016JA023601 (2017).
12. Ciraolo, L. Calibration errors on experimental slant total electron content (TEC) determined with gps. *J. Geod.* **81**, 111–120, DOI: 10.1007/s00190-006-0093-1 (2007).
13. Rawer, K., Lincoln, J. V. & Conkright, R. O. International Reference Ionosphere – IRI 79, World Data Center A for Solar-Terrestrial Physics, Report UAG-82, Boulder, Colorado (2014).
